# Compassion meditators show less anger, less punishment, and more compensation of victims in response to fairness violations

**DOI:** 10.3389/fnbeh.2014.00424

**Published:** 2014-12-09

**Authors:** Cade McCall, Nikolaus Steinbeis, Matthieu Ricard, Tania Singer

**Affiliations:** ^1^Department of Social Neuroscience, Max Planck Institute for Human Cognitive and Brain SciencesLeipzig, Germany; ^2^Mind and Life InstituteHadley, MA, USA

**Keywords:** prosocial behavior, economic games, social preferences, altruistic punishment, compassion, altruism

## Abstract

Fairness violations elicit powerful behavioral and affective responses. Indeed, people are willing to incur costs to sanction unfair behavior. Here we study the possible impact of long-term mental training in socio-affective capacities such as compassion on altruistic punishment and compensatory behavior in economic games. To this end we recruited a group of long-term meditation practitioners (LTPs) who had engaged in an average of 40 K h of mental training exercises including compassion-related meditation, along with a group of meditation-naïve controls. Participants played several adaptations of the dictator game in which they had the opportunity to punish the dictator both when they were the recipients of the dictator's offer and when they were third-party witnesses to the dictator's treatment of an anonymous second player. Compared to controls, LTPs were less likely to punish when they were the victims of fairness violations. However, both groups punished equivalently when they witnessed others receiving unfair treatment. In post-task questionnaires, controls reported significantly more anger in response to unfair offers than LTPs, although fairness judgments did not differ between groups. These data suggest that because the LTPs were less angered by unfair treatment of themselves, they punished that behavior less. However, when they witnessed the unfair treatment of others, they engaged in norm-reinforcing punishment. Finally, when participants played an additional game which included the opportunity to recompense victims, LTPs were more likely to do so. Together these data point to differential approaches to justice whereby LTPs engaged less in vengeful, retributive justice and focused more on norm reinforcement and the restoration of equity. These differences suggest that social preferences are plastic and that altruistic responses to unfairness may be shaped by the prolonged cultivation of prosocial motivation, altruism, and compassion.

## Introduction

Human social behavior is governed in part by a set of pervasive norms (i.e., Fehr and Schmidt, 1999). Particularly in the context of social exchanges, these norms influence one's decisions about how to behave toward the other parties. Fairness norms, for instance, help determine when one rewards prosocial behavior or punishes antisocial behavior. Despite the ubiquity of such norms, decisions in social situations are also subject to an individual's social preferences, such as the degree to which one cares about the well-being of others, both positively and negatively (Fehr and Camerer, 2007). These social preferences are closely tied to the emotional reactions elicited by norm violations. Accordingly, decisions made in social exchange paradigms can result from emotions elicited during an interaction. Thus, anger in response to fairness violations can be a powerful determinant of whether such violations will be subsequently sanctioned or not (Fehr and Gächter, 2002; Sanfey et al., 2003; Hopfensitz and Reuben, 2009). In theory, the social preferences that determine these responses are highly stable and trait-like (Fehr and Schmidt, 1999). But are they actually fixed and impervious to experience or learning? Or do they change and evolve within an individual over time?

To explore these questions, we studied a unique sample of long-term meditation practitioners (LTPs) who had spent years engaged in different forms of mental training intended to cultivate qualities such as loving kindness, altruism, and compassion. Compassion has been defined as the cognitive and emotional experience of concern in response to others' suffering associated with a motivation to promote the well-being of others (Dalai Lama and Vreeland, 2001; Keltner and Goetz, 2007; Fehr et al., 2009). It entails positive affect and concern for different people, including loved ones and strangers, as well as difficult persons (Salzberg, 2002). The mental training of the LTPs in this study was directed at both cognition and affect with practices focused on mental concentration, the cultivation of concern for the well-being of others, and the shifting from self- to other-oriented perspectives (Dalai Lama and Vreeland, 2001; Lutz et al., 2008; Klimecki et al., 2013b). We were interested in the extent to which this extensive training in prosocial motivation and emotions might influence affective responses to fairness violations, as well as behavioral responses toward their perpetrators and victims. Given that negative emotional responses to fairness violations bear costs both in terms of subjective well-being as well as the subsequent break-down of cooperation (Singer and Steinbeis, 2009), specific interventions might buffer the detrimental personal and interpersonal effects of unfair social interactions.

Several arguments suggest an important link between compassion and responses to fairness violations. For one, the definition of compassion as representing feelings of concern for another's suffering and the desire to increase that person's welfare (Keltner and Goetz, 2007; Fehr et al., 2009) posits a motivational state: the desire to relieve another's suffering. This definition is akin to the notion of empathic concern, a concept used widely in developmental and social psychology (Davis, 1983), which has been linked to prosocial behavior (Batson et al., 1981; Batson, 2009). For instance, participants who had received the instruction to generate empathic concern for a person receiving painful electric shocks were more willing to receive shocks themselves in order to relieve the other person of their suffering (Batson et al., 1981). A similar empathic concern induction was also shown to affect economic decision-making. In a one-shot prisoner's dilemma game, this induction led to greater cooperation with an unrelated individual compared to when empathy was not induced (Batson and Moran, 1999). More recently, inducing empathic concern was shown to buffer the negative effects of previous defection in repeated interactions (Rumble et al., 2009). In this study, participants played a repeated prisoner's dilemma game with a partner who could defect deliberately as well as accidentally (i.e., through a fault of the computer). Here the empathic concern induction led to decreased retaliation, especially when the partner's defection was accidental. Another study showed that short-term compassion training led to increased helping behavior in the context of a computer game designed to assess prosocial behavior (Leiberg et al., 2011). A further study showed that individuals who had completed meditation training were more likely to likely to help a stranger (by offering their seat to a woman on crutches; Condon et al., 2013). Finally, a study on the effects of training compassion meditation showed increased altruistic redistribution of funds to victims of fairness violations (Weng et al., 2013).

While these studies attest to the effects of empathic concern and compassion-related training on altruism and prosocial behavior, questions remain regarding how compassion training uniquely affects reactions to norm-violation such as punishment behavior (i.e., the sanctioning of perpetrators) or compensatory behavior (i.e., the compensation of victims) after unfair treatments. Furthermore, it is unclear whether training alters perception of fairness or affective responses to fairness violations, and which of these might be responsible for any observed behavioral changes. Seeing that an inherent aspect of Buddhist mental training is the cultivation of prosocial qualities such as altruism, kindness, and compassion as well as the regulation of difficult afflictive emotions, we expected LTPs to display less anger after fairness violation while still engaging in sanctioning behaviors that enforce fairness norms (Singer and Steinbeis, 2009). In broader terms, we predicted that the emotional and behavioral responses of the LTPs would reflect less retributive or vengeful justice and more restorative justice, aimed at reestablishing equity among the parties concerned (Nozick, 1981; Darley and Pittman, 2003; Greene, 2008; Aharoni and Fridlund, 2012; FeldmanHall et al., 2014).

To test for the possible effects of training in compassion and altruism on responses to fairness violations we used variants of the dictator game (e.g., Kahneman et al., 1986; Forsythe et al., 1994; Camerer, 2003), giving participants the opportunity to punish the actions of the dictator. Specifically, participants could invest their own endowment to reduce that of the dictator after either having been the recipient of an offer ranging from fair to unfair (second party), or having been the passive observer of such an interaction between two individuals (third party). In experimental game theory it has repeatedly been shown that unfair offers lead to altruistic punishment, either in the form of an offer rejection in the ultimatum game or by means of paying monetary units (MUs) in order to reduce the endowment of the one making the offer (Fehr and Gächter, 2002; Fowler et al., 2005; Henrich et al., 2006). Importantly, emotional reactions to unfair offers, such as anger, are frequently the motivating force behind altruistic punishment (Fehr and Gächter, 2002; Hopfensitz and Reuben, 2009). Seeing that compassion entails the generation of concern for the welfare of others and prosocial motivation, even toward difficult others, we hypothesized that LTPs would report less anger in response to unfair offers than controls (Singer and Steinbeis, 2009). But because the cultivation of compassion should not affect norms about what is fair and just, we did not predict any differences in perceptions of fairness. Because anger is likely to be particularly influential when individuals are the targets of unfairness and because we believed practitioners would experience less of this negative affect, we predicted that practitioners would show less of this punishment in the 2nd party dictator game. Conversely, we predicted that LTPs would demonstrate comparable punishment as the 3rd party witness to unfair offers, when they could act to reestablish equitable outcomes via norm reinforcement (Singer and Steinbeis, 2009).

In a third variant of the game, we investigated whether LTPs would also show different preferences than controls when given, in addition to punishment, the option to pay to recompense victims of fairness violations. It has previously been shown that short-term compassion training leads to redistribution of a dictator's funds after having made an unfair offer in order to equalize the pay-off between the two players (Weng et al., 2013). The implementation of equalizing in that study occurred by means of punishing the dictator, therefore confounding the desire to equalize with the desire to punish. By giving our participants the opportunity to recompense either other player in a third party dictator game, as well as to punish them, we could now dissociate these two different motives. Here we hypothesized that when given the opportunity, LTPs would show a greater tendency than controls to restore justice by spending money to compensate individuals who have been treated unfairly.

In sum, we expected our LTPs, in comparison to the control group, to (a) show less anger in response to fairness violations, (b) show less second-party altruistic punishment, (c) similar degrees of third-party altruistic punishment, and (d) more recompesatory behaviors toward the victims of fairness violations.

## Methods

### Participants

The study involved two groups of participants: LTPs and controls. The 18 LTPs (6 women, ages 45–62, mean ± SD age = 54.3 ± 5.8 years) were practitioners of the Nyingma tradition of Tibetan Buddhism, a practice focused on loving-kindness, altruism, and compassion. Each had participated in a full-time meditation retreat of three or more years at the Songsen Chanteloube Retreat Center in Dordogne, France. The LTPs were recruited as part of a larger study on the effects of prolonged mental training on a variety of domains including attention, pain, working memory capacity, emotional reactivity, as well as brain function and brain structure. They were contacted directly via email and asked if they would be willing to participant in a series of studies on the effects of mental training on the mind and brain. In addition, we measured 15 age-matched controls (5 women; 46–63 years, mean ±SD age = 54.3 ± 5.8 years). We assessed the IQ of all subjects using Raven's Progressive Matrices (Raven, 2000). LTPs and controls did not differ with respect to IQ (103.6 ± 20.0 vs. 102.4 ± 28.5). All participants provided informed consent to participate in the study and were paid for their participation. The study was approved by the University of Leipzig's Ethics Committee. All participants took part in extensive tests over a 2 day period including brain structure and function, emotion regulation, sustained attention and attentional reactivity, task-independent thought as well as a range of questionnaires. Whereas, these will not be reported here, given the comprehensiveness of psychological assessments over a wide range of domains it is likely that experimenter demands for any single set of experiments were considerably reduced. In addition to a normal participation fee for participation across all experiments, participants were also paid an additional sum of money dependent on their decisions made in the games. Thus, decisions were financially incentivized. Each MU in the game was translated into one cent. At the end all MUs retained by the participant were paid out.

### Games

**Figure 1:**
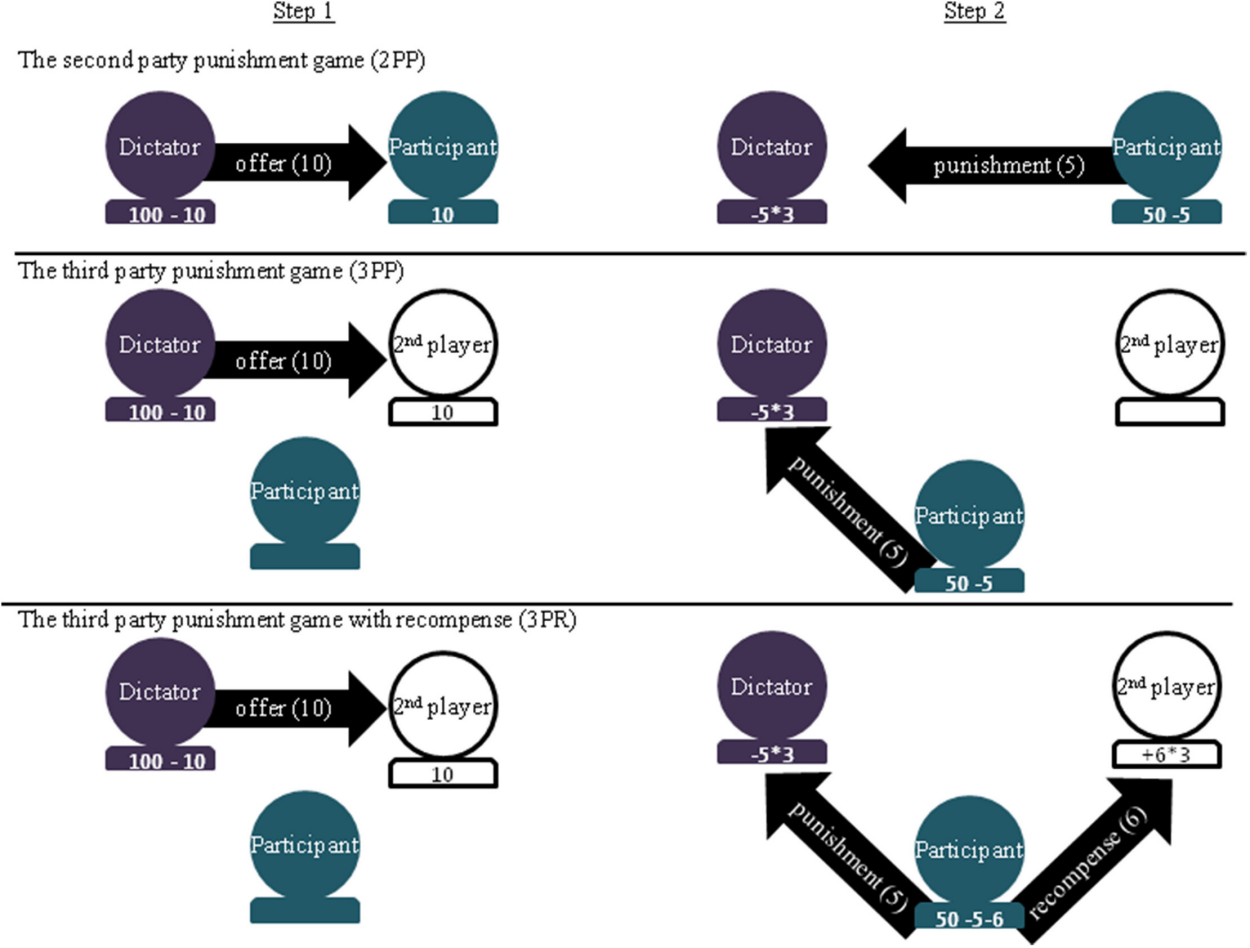
**Schematics for the three different economic games with examples of monetary exchanges for each step**. In the 2PP, participants received offers from the dictator (e.g., 10 MUs) and then, in the second step of the game, had the option to pay to punish the dictator (e.g., paying 5 to deduct 15 MUs). In both the 3PP and the 3PR, participants witnessed the dictator send an offer to the 2nd player and could again pay to punish the dictator. In the 3PR, participants had the additional option of paying to recompense the 2nd player (e.g., paying 6 to recompense 18 MUs).

Participants were tested in individual testing sessions which lasted approximately 1.5 h and were conducted in a computer lab at the Max Planck Institute for Human Cognitive and Brain Sciences in Leipzig, Germany. Participants completed three separate economic games over the course of an approximately 1.5 h experimental session (see Figure 1): a dictator game (e.g., Kahneman et al., 1986; Forsythe et al., 1994; Camerer, 2003) with second-party punishment (2PP), a dictator game with third-party punishment (3PP), and a dictator game with third party punishment and recompense (3PR). The games were completed on a desktop computer and were custom programmed in the Python scripting language. The details of each of these games are provided below. Before each game, participants received detailed instructions explaining the rules. The instructions for each of these games are included in the Supplementary Materials. After reading these instructions, they completed a series of questions designed to test their understanding of the game. If participants made any mistakes on this “quiz,” the experimenter went back over the instructions verbally and asked the participant to correct their answers. This procedure was repeated until participants provided all correct responses. Because of time constraints, one participant did not complete the 3PR and a second participant did not complete one trial of the 3PR.

To determine which role in the game participants were supposed to play, we pretended to draw lots. These lots led to our participants always being in the role of the person who was able to sanction norm violations. For each game, the investment behavior of the dictator was determined by the computer. However, we led participants to believe that they were interacting with actual people via an online platform which networked participants from across Europe (see also Singer et al., 2004 for similar procedures). To create the illusion of this “European Consortium,” waiting periods of random lengths (2–8 s) were interspersed between rounds of each game during which the system ostensibly waited for another player on the network to join the next round. Participants completed 13 rounds of each game and they were led to believe that these rounds were one-shot, i.e., that they would not encounter the other player again. This manipulation was crucial given the scarcity of the LTPs and the fact that for the two groups of subjects to be comparable to one another in terms of their decisions. All participants were fully debriefed of the intention and manipulation of the study afterwards.

The first round of each game was that of a classic dictator game (Camerer, 2003). The first player (i.e., the confederate) was allotted 100 MUs and ostensibly given the option to share some of those MUs with the second player. First plays ranged from 10 to 50 MUs with the first player investing 10, 30, and 50 MUs on three occasions each and 20 and 40 on two occasions. In the 2PP the participant was the second player and, as such, received this offer. In the 3PP and 3PR the participant was a third-party observer to an interaction between two participants who were playing on-line.

In the 2PP and 3PP, participants were given the opportunity to punish the first player. They were allotted a total of 50 MUs and told that for every 1 MU they spent, three times that value would be deducted from the first player's earnings for that round. Punishment was chosen using a slider on the game interface.

In the 3PR, participants were again given 50 MUs to punish or recompense each of the players, with each MU spent either detracting or adding to the other player's pot. For this game, the participant was given a slider for each of the other players. The slider could be moved to the left of center to punish and to the right of center to recompense. For example, if a participant moved the first player's slider 2 MUs to the left, 6 MUs would be deducted from the first player's pot. And if a player moved the second player's slider 2 MUs to the right, the second player would receive 6 additional MUs.

Participants completed the 2PP and 3PP first and the order of these games was counterbalanced. Participants always completed the 3PR game last. Throughout the reported analyses, we use linear mixed models to test the effects of group (LTP vs. control), game, and dictator offer on behavior while accounting for subject level random effects.

### Questionnaires

After the games, participants completed a short series of questionnaires. In one questionnaire, participants rated their emotional responses to the five first offers made by the dictators during the games. On a Likert scale ranging from “Not at all” to “Very,” participants rated how angry, sad, happy, or disgusted they felt after receiving the offer. Participants answered these questions twice: once for their experience as the second player (in the 2PP) and once for their experience as the third player (in the 3PP and 3PR). Participants also answered more general questions about perceptions of fairness. In this questionnaire (Steinbeis et al., 2012) they were presented with a series of different distributions of MUs between two people (e.g., one person gets one and the other gets nine, one person gets five and the other gets five, and etc.). They rated the fairness of each distribution by marking a line on a continuous Likert scales. Because of time constraints, five LTPs and one control did not fill out the self-related anger questionnaires, three LTPs and one control did not complete the other-related anger questionnaire, and 3 LTPs and one control did not complete the fairness questionnaire.

## Results

### Dictator game with second party punishment

**Figure 2:**
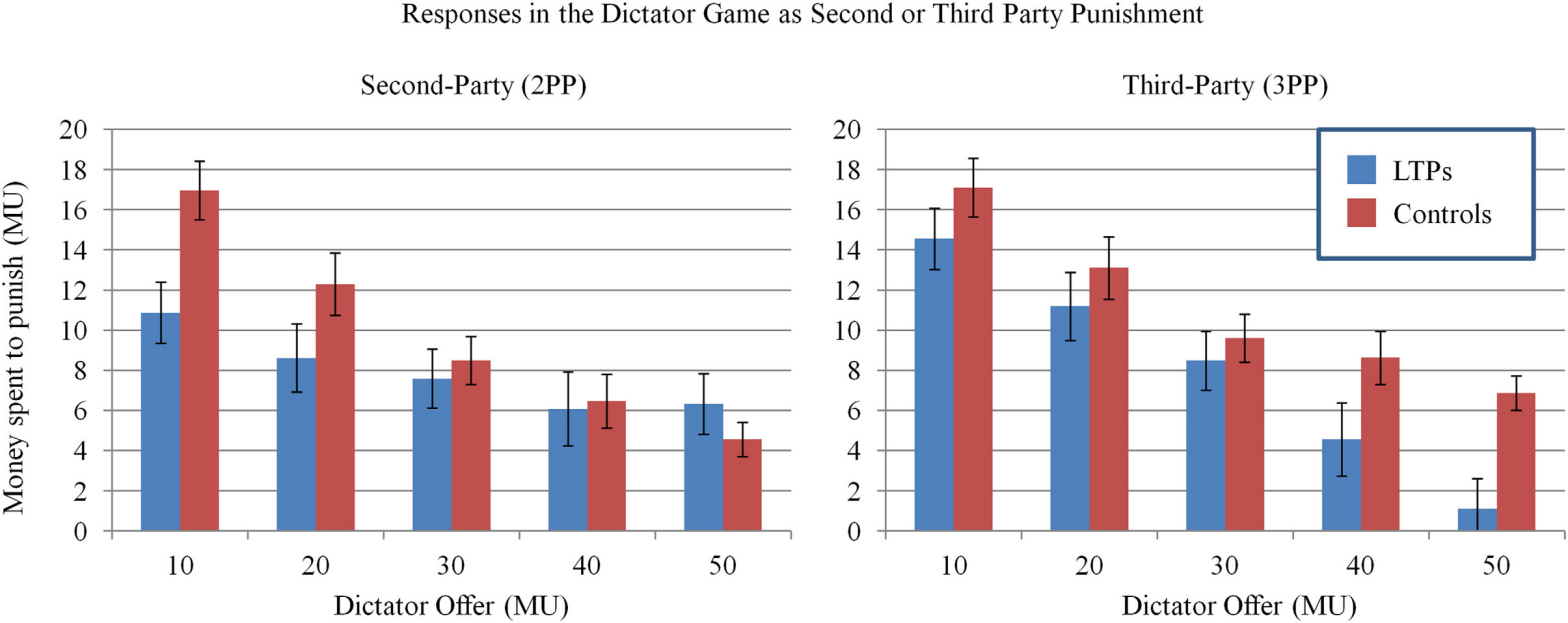
**Plots of the pooled means and their standard errors from the dictator games with second and third-party punishment (2PP and 3PP)**. Punishment is measured as amount spent in MUs to punish the dictator. The mixed model of punishment behavior revealed that punishment increased with decreasing size of the dictator's offer (*p*s < 0.001). LTPs punished significantly less than controls in the 2PP (*p* = 0.01) and equivalently in the 3PP. LTP punishment patterns were more strongly associated with the dictator offer in the 3PP (*p* = 0.001).

We ran a linear mixed model in SPSS 22 to predict punishment in the 2PP. The dependent variable here was MUs spent to punish on each given round. As fixed effects we entered the dictator's offer for the round (from 10 to 50 MUs), the participant group (i.e., control vs. LTP), and the interaction between these two. For subject-related random effects we entered the dictator's offer and intercept. Because of the distribution of punishment values (i.e., strongly positively skewed with a mode of zero), we used a negative binomial probability distribution with a log link in the model. In line with the hypothesis, this model revealed a significant effect of group whereby LTPs punished significantly less than controls, *p* = 0.01 (see Table 1 for beta values and Figure 2 for illustration). There was also a significant effect of dictator offer whereby participants punished more for smaller (and thus more unfair) offers, *p* < 0.001. The interaction between group and dictator offer was not significant. The betas for the individual predictors and their standard errors are reported in Table 1,^1^. Supplementary Table 1 further reports proportion of trials in which punishment occurred as well as the number of participants who chose to punish for this and the subsequent games.

**Table 1:** **Beta values and standard errors the four linear mixed models predicting punishment behavior in the 2PP, 3PP, and 3PR as well as recompense behavior in the 3PR**.

	Second party punishment (2PP)		Third party punishment (3PP)		Punishment and recompense (PR)			
					Punishment		Recompense	
	***b***	***SE***	***b***	***SE***	***b***	***SE***	***b***	***SE***
Intercept	3.2^***^	0.55	2.85^***^	0.51	−14.7^***^	2.7	8.1^**^	2.63
Dictator Offer	−0.054^***^	0.01	−0.03^***^	0.01	0.32^***^	0.04	−0.149^***^	0.04
LTP (group)	−1.88^*^	0.76	−0.11	0.69	3.5	3.7	6.7°	3.6
Dictator Offer × LTP	0.02	0.02	−0.04^***^	0.01	−0.1	0.06	−0.09^*^	0.05

Each model used the size of the dictator's offer, the participant group (LTP vs. control) and their interaction as predictors. The model for punishment in the 2PP (F = 11.5^***^, observations = 429), punishment in the 3PP (F = 32.3^***^, observations = 429), punishment in the 3PR (F = 32.1^***^, observations = 415), and recompense in the 3PR (F = 25.4^***^, observations = 415) were each significant

(* p < 0.05,

** p < 0.01,

*** p < 0.001,

° pone-tailed < 0.05).

### Dictator game with third party punishment

We ran another linear mixed model with the same structure to predict punishment in the 3PP (see Table 1 and Figure 2). In this game, there was no significant difference between group, *p* = 0.87. There was again the effect of dictator offer on punishment, *p* < 0.001, whereby participants punished more for less fair offers. There was also an interaction with this variable and group, *p* = 0.001 whereby LTPs increased their punishment more strongly to more unfair offers.

Because different punishment patterns emerged between LTPs and controls in the 2PP and 3PP, we ran additional analyses to test for interactions between the games and group (Tables for these models provided in the Supplementary Materials, Supplementary Tables 2, 3). In this mixed model, punishment was the dependent variable. Game (2PP vs. 3PP), group, dictator offer, and the interactions between these variables were entered as fixed effects. Subject level random effects were again the level of the dictator offer and the intercept. Indeed, significant interactions emerged between dictator offer and group, β = −0.037, *SE* = 0.014, *p* = 0.006, between game and group, β = −1.72, *SE* = 0.50, *p* = 0.001, as well as game and group and dictator offer, β = 0.06, *SE* = 0.015, *p* < 0.001. To probe the effect of game further, we analyzed each group separately. In these models, the fixed effects were game and dictator offer and the interaction between the two. Subject-level random effects were the dictator offer (the model was unable to converge with the intercept was included as a random effect so it was excluded from these models). When we ran this model to look at LTP behavior, dictator offer, was significant, β = −0.145, *SE* = 0.039, *p* < 0.001. More importantly, the effect of game was significant whereby LTPs punished more in the 3PP game than in the 2PP game, β = −1.22, *SE* = 0.42, *p* = 0.003. The interaction between game and dictator offer was also significant, revealing that LTP punishment was more strongly related to the decreasing fairness of the dictator offer in the 3PP, β = 0.035, *SE* = 0.013, *p* = 0.008^2^. Finally, we ran this model on the control subjects. Although dictator offer had its usual effect of increasing punishment with decreasing fairness of dictator offers, β = −0.048, *SE* = 0.016, *p* = 0.003, there was no effect of game or of the interaction between game and offer (*p* = 0.70 and *p* = 0.11, respectively).

### Dictator game with third party punishment and recompense

**Figure 3:**
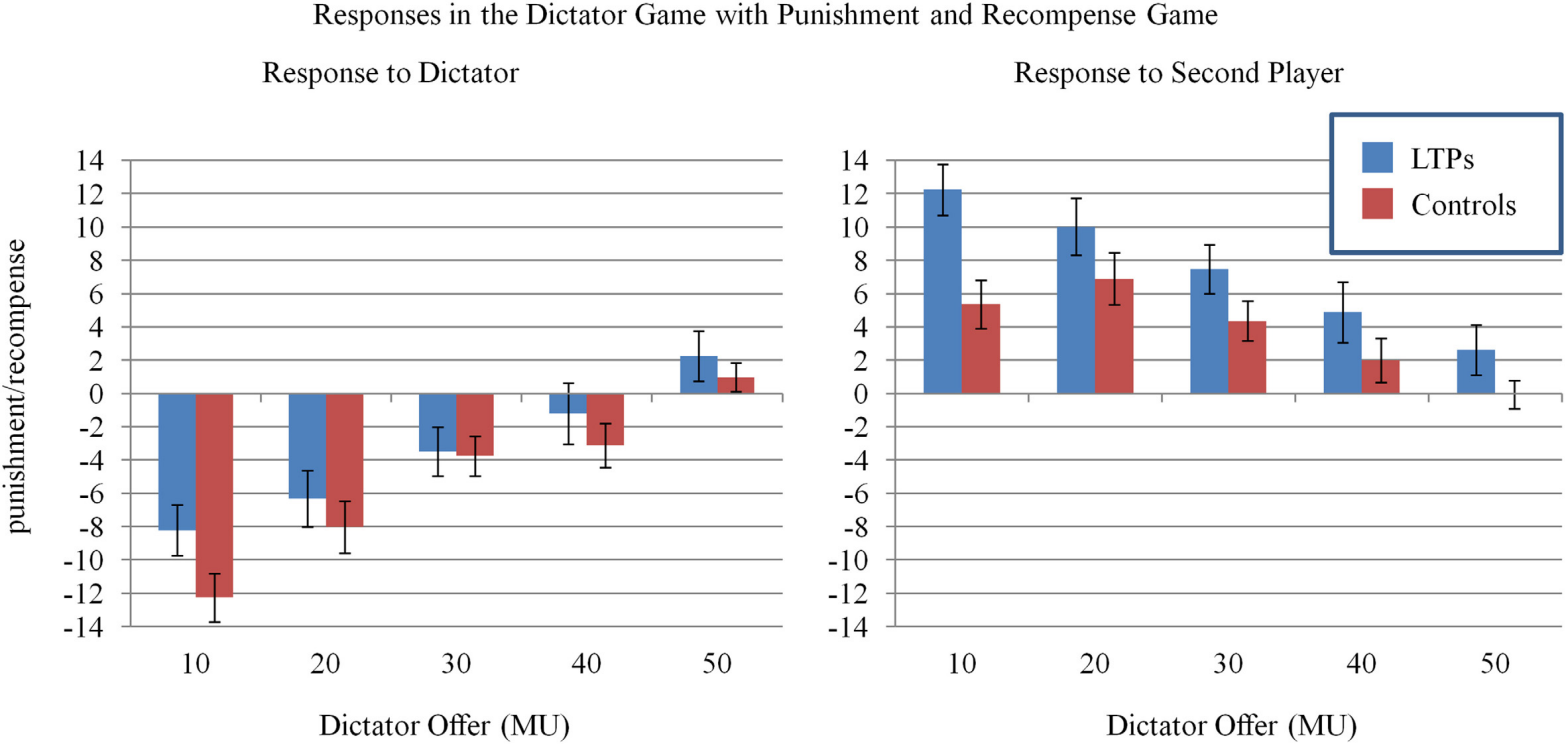
**Plots depicting pooled means and standard errors of responses in the dictator game with third-party punishment and recompense (3PR)**. Punishment is represented as a negative number where one MU spent to punish is −1 MU. Recompense is represented as a positive number where one MU spent to recompense is +1 MU. The mixed models of these data revealed that as dictator offers decreased, participants punished the dictators and recompensed the second players more (*p*s < 0.001). LTPs' punishment behavior did not differ from controls, but they were more likely to recompense (*p*_onetailed_ = 0.03). Moreover, LTPs' recompensatory behavior was more strongly coupled with Dictator Offers (*p* = 0.05).

To examine participant responses in the 3PR, we ran two more linear mixed models, one focusing on the response to the dictator and the other focusing on the response to the second player. The difference from the previous models was the dependent variable which was, this time, the number of MUs spent on to either subtract or add to the player's endowment. To distinguish within this variable between subtracting (i.e., punishing) and adding (i.e., recompensing), subtracting was coded as a negative value and adding was coded as a positive value. Because these variables were normally distributed than in the previous games, we used a normal probability distribution and identity link function to the model. We first ran this model on responses to the dictator and found a similar pattern to the one that emerged in the 3PP (see Table 1 and Figure 3). Participants punished more for less fair dictator offers, *p* < 0.001. There was neither an effect of group nor any interaction between group and dictator offer, *F*s < 1.

To test the hypothesis that LTPs would be more likely to recompense victims of unfairness violations, we used the same model again, this time to predict responses to the second player (see Table 1 and Figure 3). There was a main effect of dictator offer whereby participants were more likely recompense the second player as the dictator's offers decreased in fairness, *p* < 0.001. There was also the main effect of group of group in the predicted direction whereby LTPs recompensed more, *p*_one−tailed_ = 0.03. Furthermore, an interaction emerged between group and dictator offer in the predicted direction such that LTPs showed a steeper response to unfairness, increasing recompense in line with the decreasing fairness of dictator offers, *p* = 0.05.

### Emotional responses to unfair offers

To evaluate the degree to which the different dictator offers elicited emotional responses and to test our hypothesis that LTPs would respond to unfairness with less anger, we ran a series of repeated measures ANOVAs on participants' post-task reports of feeling angry, sad, disgusted, or happy when either receiving an offer of a given size in the 2nd party game (2PP) or when witnessing the second party receive that offer in the 3rd party games (3PP and 3PR). For each of these models, group (LTP vs. control) was a between-subjects predictor and dictator offer (10 through 50 by increments of 10) was a within-subjects predictor. The rating of the specific emotion was the dependent variable. We ran this model for each emotion (anger, sadness, disgust, and happiness) and for each type of second player (i.e., when the participant was the 2nd player or the 3rd player). The ANOVAs for anger produced several significant results. When participants were the 2nd party recipients of dictator offers, there was a main effect of anger whereby participants were angrier the smaller the offer, *F*_(4, 22)_ = 5.6, *p* = 0.003. There was also the significant effect of group in the predicted direction whereby LTPs were less angry than the controls, *F*_(1, 25)_ = 10.1, *p* = 0.004. Further, a significant interaction between the two predictors emerged, *F*_(4, 22)_ = 3.5, *p* = 0.023, revealing a weaker slope in the relationship between offer size and anger among LTPs. The predicted pattern of less anger among LTPs also emerged for anger responses when participants were the 3rd party witnesses. This ANOVA again revealed significant effects of offer size, *F*_(4, 24)_ = 3.7, *p* = 0.02 and the hypothesized effect of group in the predicted direction, *F*_(1, 27)_ = 4.0, *p*_onetailed_ = 0.03 and interaction between group and offer size in the predicted direction *F*_(4, 24)_ = 2.6, *p*_onetailed_ = 0.03. We repeated these same analyses for sadness, disgust, and happiness in the 3rd and 2nd party games. No significant effects (or interactions with) group emerged (all *p*s > 0.30).

### Fairness evaluations

**Figure 4:**
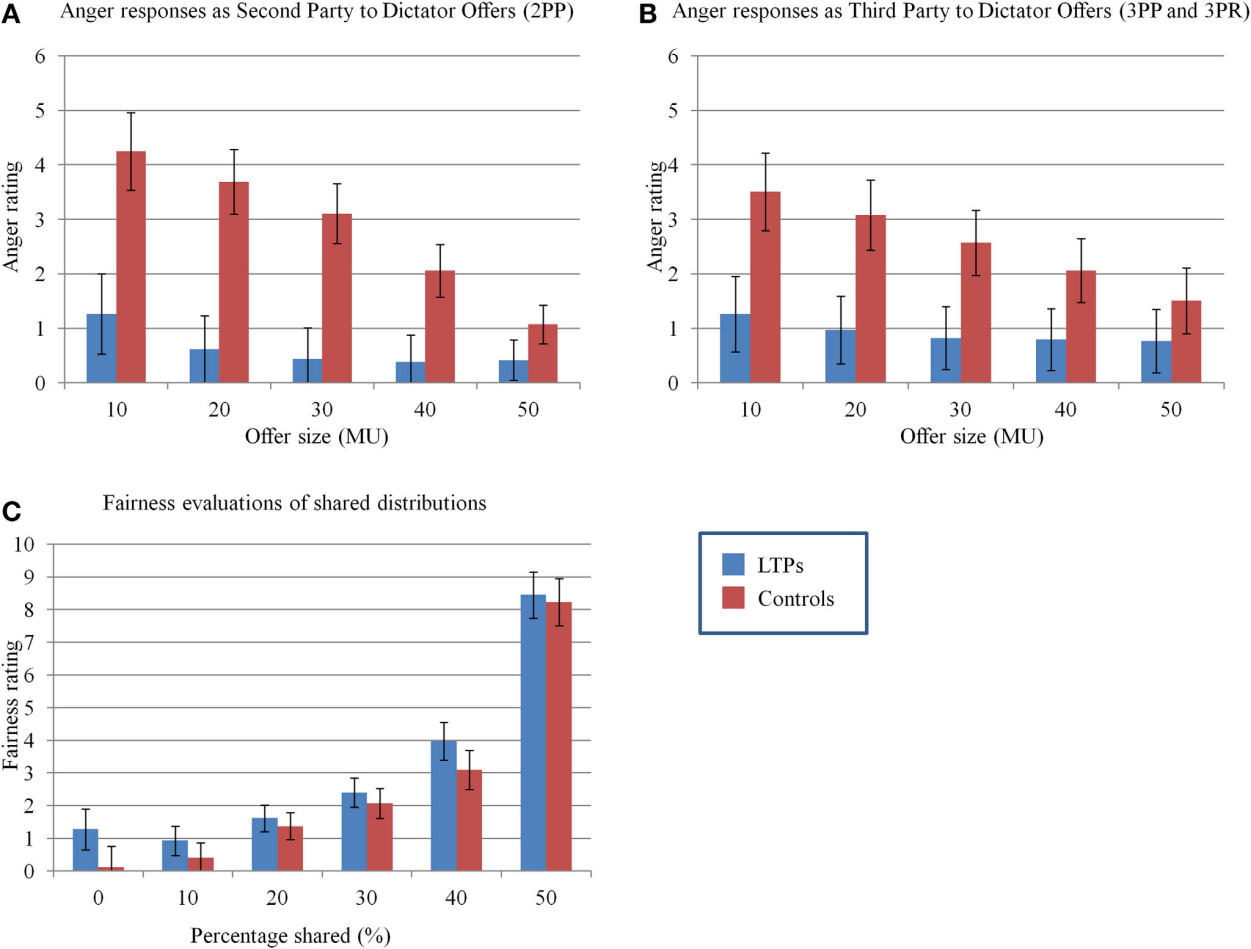
**Estimated marginal means and their standard errors for anger responses to being the second player (A) or third party witness (B) to a range of dictator offers as well as fairness ratings of a range of monetary distributions (C)**. LTPs were significantly less angry than controls when they were either the 2nd (*p* = 0.004) or 3rd party (*p*_onetailed_ = 0.03). However, they held equivalent evaluations of fairness.

To compare fairness norms between groups, we ran a repeated measures ANOVA on participant responses on the fairness questionnaire, using group as a between-subjects predictor and distribution level as a within-subjects predictor (Figure 4). We found a significant effect of distribution level, *F*_(5, 23)_ = 25.2, *p* < 0.001, revealing that more unequal the distribution, the less fair participants' ratings. More importantly, however, there was no effect of group, *F*_(1, 27)_ < 1.6, suggesting that although groups differed in the amount of anger expressed this was not due to different perceptions of fairness.

### Anger, fairness, and punishment

Next, we analyzed the relationship between self-reported anger toward dictator offers and actual punishment behavior. To address this question we ran a mixed model in which the dependent variable was the punishment behavior in the 2PP and 3PP games, again with a log link to a negative binomial distribution. The predictors were group (LTP or control), game (2PP or 3PP), and the reported anger for the given offer size and second player (i.e., self vs. other), and the interactions between these variables. Subject intercept was entered as a random effect. This model produced a significant effect of anger whereby anger predicted punishment, β = 0.18, *SE* = 0.05, *p* < 0.001. The beta values for group and for the interactions were not significant (Supplementary Table 4). We used a similar model to predict punishment in the 3PR, using a linear distribution for punishment (as above when predicting punishment in the 3PR). The predictors in this model were group (LTP or control), reported anger for the given offer size, and their interaction. This model replicated the findings from the 2PP and 3PP with anger predicting greater punishment, β = −1.76, *SE* = 0.27, *p* < 0.001. Again, there were no significant betas for group or the interaction (Supplementary Table 5).

Finally, we analyzed the relationship between fairness evaluations and punishment behavior. To do so, we ran a mixed model in which the dependent variable was punishment in both the 2PP and 3PP, with a log link to a negative binomial distribution. The predictors were group, game, and fairness evaluation for the given offer size, and the interactions between these variables. Subject intercept was entered as a random effect. This model revealed a significant effect of fairness in which the less fair participants evaluated an offer, the more they punished the dictator, β = −0.10, *SE* = 0.04, *p* = 0.005. As one would expect from the previous analyses, there was an interaction between group and game, reflecting the fact that LTPs punished less in the 2PP, β = −0.937, *SE* = 0.346, *p* = 0.007. There was also an interaction between fairness evaluations and group, whereby the relationship between fairness evaluations and punishment was stronger in LTPs, β = −0.181, *SE* = 0.05, *p* = 0.001. This stronger relationship between fairness evaluations and punishment was driven by behavior in the 3PP, as is demonstrated by a significant interaction between fairness evaluations, game, and group, β = 0.27, *SE* = 0.07, *p* < 0.001 (Supplementary Table 6). We ran an additional model to see if these effects replicated punishment in the 3PR, using a linear distribution for punishment. The predictors in this model were group (LTP or control) and fairness evaluations for the given offer size and their interaction. Subject intercept was entered as a random effect. Fairness again predicted punishment, β = 1.17, *SE* = 0.13, *p* < 0.001, although the interaction did not emerged as significant (Supplementary Table 7).

## Discussion

In this study, we examined behavioral and affective responses to fairness violations in several economic games among a unique sample of individuals with long-term training in the cultivation of prosocial qualities such as altruism, compassion, and prosocial motivation. Given the nature of such training, we expected these long-term practitioners to demonstrate less anger in response to unfairness than gender- and age-matched controls. By consequence we expected them to show comparatively less punishment than controls when they were the direct victims of fairness violations, but comparable norm-reinforcement through altruistic punishment when they were third-party witnesses to injustice. Finally, we expected more compensatory behaviors when participants were given the option to restore justice by giving money to the victims of unfairness.

As predicted, the results revealed differential patterns between LTPs and controls in response to a series of dictator games with options to punish and recompense. In line with previous reports in the literature (Fehr and Gächter, 2002; Sanfey et al., 2003), both groups of subjects increased altruistic punishment when dictator offers grew increasingly unfair. As expected, the extent of punishment among the LTPs was reduced when they themselves were the victims of unfairness (in the 2PP game), but comparable to controls when they were 3rd parties merely witnessing an unfair interaction between two other players (in the 3PP and 3PR games). Although LTPs and controls did not differ in their beliefs about fairness, unfair behavior elicited less anger from the LTPs. Given that anger and fairness evaluations both generally predicted punishment in this study and in the literature (Fehr and Gächter, 2002; Hopfensitz and Reuben, 2009), this pattern of results suggests that the long-term practitioners punished less when they were 2nd party recipients of unfair offers because they were less angry, but were driven by fairness norms to punish equally when they were 3rd parties witnesses to injustice. In line with such an interpretation, the altruistic punishment behavior of the LTPs in the 3PP was more tightly coupled to both the actual level of dictator offers and to their subjective evaluations of the fairness of those offers.

Nevertheless, this pattern of results raises the question as to why the LTPs did not punish equivalently in the 2PP even though, in general, fairness predicts punishment. The current data do not allow for a satisfying answer, although they parallel recent findings that the punishment of fairness violations, as well as the compensation of victims of those violations, depends in part on whether the target of the fairness violation is the self vs. another person (FeldmanHall et al., 2014). Further work will need to more deeply probe the motivational distinction between acting on behalf of oneself vs. on behalf of another to better understand the mechanisms underlying these differences. Along similar lines, other motives underlying punishment behavior have been suggested in the literature, such as spite (Jensen, 2010; Espin et al., 2012; Brañas-Garza et al., 2014). While it may be possible that the present data could also in part be explained by the operation of other mechanisms (i.e., the presence of spite or responses to inequality), our evidence speaks most clearly for a motivation of anger as a result of unfairness to underlie the punishment behavior of both LTPs and controls, a finding in line with previous studies (Fehr and Gächter, 2002). Future studies should focus on exploring the plasticity of other social motivations in the context of altruistic punishment.

This focus on equity among the LTPs emerged again in the 3PR when participants were given the opportunity to recompense victims in addition to punishing perpetrators of fairness violations. Whereas, it has previously been shown that a short-term intervention of compassion meditation in naïve subjects increased the tendency to redistribute funds between an unfair dictator and his victim (Weng et al., 2013), it remained an open question whether this was the result of a desire to punish the dictator or to recompense the victim. Here we disentangled these motives by providing participants with both the opportunities to punish and recompense the players. Although LTPs again punished in a similar fashion to controls, they showed a distinct pattern of recompensation, increasing their donation to the victim as the dictator's offers became progressively less fair. These data suggest that the cultivation of altruism and compassion may selectively increase the motivation to perform a kind act toward a victim of a transgression and not the merely desire to punish *per se*.

Previous studies have shown that anger in response to fairness violations and subsequent punishment behavior are linked (Fehr and Gächter, 2002; Hopfensitz and Reuben, 2009; Grecucci et al., 2013). Together, our results indicate that training feelings of compassion may alter behavioral responses to fairness violations through reducing these concomitant negative emotions experienced when responding directly to unfairness, while still supporting the motivation to minimize inequity (see also Singer and Steinbeis, 2009). Accordingly, two studies by Klimecki et al. (2013a,b) showed that training in compassion selectively increased activity of brain regions known to be involved in positive affect, such as the ventral striatum and the medial orbitofrontal cortex, when confronted with the suffering of another. We speculate that increased prosocial motivation and affect in response to the suffering of others can serve as a buffer in social situations which, in the present case, reduced anger in response to unfair offers and led to increased motivation to restore equity by means of compensating the victim.

These findings have important implications for the evaluation and enforcement of justice. The fact that LTPs punished in the relative absence of anger suggests that training in compassion can lead from sanctioning as a function of vengeful and retributive motives (i.e., punishment to punish the transgressor) to sanctioning in order to restore justice and equity (i.e., to solve the problem). This potential shift from a more deontological to a more consequentialist approach to punishment would represent a shift from common lay responses to injustice that are reactionary and potentially counterproductive to a sharper focus on the actual outcomes of punishment behavior itself (Nozick, 1981; Darley and Pittman, 2003; Greene, 2008; Aharoni and Fridlund, 2012). Indeed, it has previously been shown that whereas punishment in social interactions is effective to sustain cooperation, it also incurs hidden costs to all parties involved, particularly over repeated interactions (e.g., Fehr and List, 2004; Rand et al., 2009). The fact that LTPs were uniquely concerned with compensating victims further argues that their focus was, more broadly, on an equitable outcome and not merely on vengeance for bad behavior. Furthermore, given evidence that the experience of sustained negative affect constitutes a reliable predictor for developing health risks (Pressman et al., 2013), showing that justice can still be done without negative emotions such as anger highlights potential health benefits of compassion training as a buffer to antagonistic social situations (see also Singer and Steinbeis, 2009).

Our findings of differences between the LTPs and controls in terms of experienced emotions and concomitant punishment and compensatory behaviors in response to fairness violations need to be carefully interpreted with regards to a potential self-selection bias of the sample. Indeed, the present study is not in a position to tease apart whether such differences arise as a function of the training or shared dispositions between choosing such trainings and our observed behaviors. However, since previous studies have been able to show that even short-term interventions of compassion training can elicit similar behaviors as observed in the present study (Weng et al., 2013), the present study can be seen as a real-life example of compassion training, which in combination is in a position to triangulate on the same point, namely the plasticity of social preferences.

Our findings must further be discussed within a more general framework of the plasticity of prosocial motivation, behavior, and social preferences. While most training studies in empathy and compassion research have so far focused on how mental training in social emotions and motivation might impact changes in brain responses associated with affiliation and reward (Klimecki et al., 2013a,b) or compassion-based helping behavior (Leiberg et al., 2011), sanctioning behaviors have not been studied (but see Weng et al., 2013). In the current research, we show that the long-term training of qualities such as altruism, compassion, and loving kindness correlate with unique patterns in norm reinforcing altruistic punishment and the recompensation of victims of unfairness. These potentially training-related changes in social preferences contradict current economic models of human social behavior, which posit a trait-like stability to preferences and suggest that they are imperviousness to influence (Becker, 1976; Stigler and Becker, 1977). Moreover, they illustrate that individuals might learn to punish injustice and help victims in the relative absence of anger, findings that have considerable ramifications for the plasticity of individual preferences and the benefits of mental training for the society as a whole.

### Conflict of interest statement

The authors declare that the research was conducted in the absence of any commercial or financial relationships that could be construed as a potential conflict of interest.

## Footnotes

^1^For this and each of the subsequent models predicting punishment/recompense behavior we re-ran the model, including sex and age as factors. Neither of these factors were significant in these models nor did their presence in the models change the pattern or significance level of results.

^2^We re-ran this model with years of meditation practice as an additional factor. This factor did not predict any difference in behavior nor did it significantly affect the results of the model, a fact that is likely attributable to the relatively number of hours practiced across all of the LTPs.
