# Supplementary material for: Compassion meditators show less anger, less punishment, and more compensation of victims in response to fairness violations
Source: Front Behav Neurosci. 2014 Dec 9;8:424. doi: 10.3389/fnbeh.2014.00424 (PMC4260514; doi:10.3389/fnbeh.2014.00424)
Supplement: Supplementary file 1 [file Table1.DOCX]

**Supplementary Tables**

**Supplementary Table 1**

|  | Proportion of trials with punishment/ recompense | |  | Number of participants who punished/ recompensed | |
| --- | --- | --- | --- | --- | --- |
|  | LTPs | Controls |  | LTPs | Controls |
|  |  |  |  |  |  |
| punishment in 2PP | 0.51 | 0.74 |  | 14 | 14 |
|  |  |  |  |  |  |
| punishment in 3PP | 0.59 | 0.81 |  | 15 | 14 |
|  |  |  |  |  |  |
| punishment in 3PR | 0.48 | 0.6 |  | 13 | 15 |
|  |  |  |  |  |  |
| recompense in 3PR | 0.63 | 0.55 |  | 14 | 15 |

Supplementary Table 1: Proportions of trials during which LTPs and Controls punished and/or recompensed as well as the number of participants who did so during the dictator games with second-party punishment (2PP),third party-punishment (3PP) or third party punishment and recompense (3PR).

**Supplementary Table 2**

|  | *b* | *SE* |
| --- | --- | --- |
|  |  |  |
| Intercept | 2.91*** | .562 |
|  |  |  |
| Dictator Offer | -.035*** | .009 |
|  |  |  |
| Game (2PP) | -.014 | .35 |
|  |  |  |
| Group (Control) | -.088 | .77 |
|  |  |  |
| Group x Game | -1.72** | .50 |
|  |  |  |
| Dictator Offer x Game | -.014 | .011 |
|  |  |  |
| Dictator Offer x Group | -.037** | .014 |
|  |  |  |
| Dictator Offer x Group x Game | .06*** | .015 |

Supplementary Table 2: Beta values and standard errors from the linear mixed models using game (2PP versus 3PP), group (LTP versus Control) and the size of the dictator offer to predict participant punishment behavior in the 2PP and 3PP games. The full model was significant (F = 13.17***, observations = 858; ** = p < .01, *** = p < .001 ).

**Supplementary Table 3**

|  | LTPs | |  | Controls | |
| --- | --- | --- | --- | --- | --- |
|  |  |  |  |  |  |
|  |  |  |  |  |  |
|  | *b* | *SE* |  | *b* | *SE* |
|  |  |  |  |  |  |
| Intercept | 3.8*** | .301 |  | 3.3*** | .188 |
|  |  |  |  |  |  |
| Dictator Offer | -.145*** | .039 |  | -.048** | .016 |
|  |  |  |  |  |  |
| Game (2PP) | -1.22** | .416 |  | .102 | .265 |
|  |  |  |  |  |  |
| Dictator Offer x Game | .035** | .013 |  | -.013 | .008 |

Supplementary Table 3: Beta values and standard errors from the two linear mixed models (one for LTPs and one for controls) using game (2PP versus 3PP) and the size of the dictator offer to predict participant punishment behavior in the 2PP and 3PP games. The full models for both LTPs (F = 6.45***, observations = 468) and controls (F = 6.59***, observations = 390) were significant ( ** = p < .01, *** = p < .001 ).

**Supplementary Table 4**

|  | *b* | *SE* |
| --- | --- | --- |
|  |  |  |
| Intercept | 1.60** | .507 |
|  |  |  |
| Target (self) | -.33 | .216 |
|  |  |  |
| Anger | .18*** | .050 |
|  |  |  |
| Group (LTP) | -.31 | .725 |
|  |  |  |
| Target*Anger | 0.04 | .052 |
|  |  |  |
| Target*Group | -0.14 | .300 |
|  |  |  |
| Anger*Group | 0.24 | .152 |
|  |  |  |
| Anger*Target*Group | 0.08 | .170 |

Supplementary Table 4: Beta values and standard errors from the linear mixed model using target (self versus other), self-reported anger about the given dictator offer, and participant group (LTP versus controls) to predict participant punishment behavior in the 2PP and 3PP games. The full model was significant (F = 6.47***, observations = 702; ** = p < .01, *** = p < .001 ).

**Supplementary Table 5**

|  | *b* | *SE* |
| --- | --- | --- |
|  |  |  |
| Intercept | -1.7 | 2.4 |
|  |  |  |
| Anger | -1.8*** | .27 |
|  |  |  |
| Group (LTP) | -1.7 | 3.4 |
|  |  |  |
| Anger*Group | -.25 | .77 |

Supplementary Table 5: Beta values and standard errors from the linear mixed model using self-reported anger about a given dictator offer and participant group (LTP versus control) to predict punishment behavior in the 3PR game. The full model was significant (F = 17.5***, observations = 350; *** = p < .001).

**Supplementary Table 6**

|  | *b* | *SE* |
| --- | --- | --- |
|  |  |  |
| Intercept | 2.3*** | .624 |
|  |  |  |
| Target (self) | -.09 | .227 |
|  |  |  |
| Fairness | -.10** | .035 |
|  |  |  |
| Group (LTP) | -.67 | .877 |
|  |  |  |
| Target*Fairness | -.05 | .049 |
|  |  |  |
| Target*Group | -.94** | .346 |
|  |  |  |
| Fairness*Group | -.181** | .054 |
|  |  |  |
| Fairness*Target*Group | .267*** | .073 |

Supplementary Table 6: Beta values and standard errors from the linear mixed model using target (self versus other), self-reported fairness evaluations for the given dictator offer, and participant group (LTP versus controls) to predict participant punishment behavior in the 2PP and 3PP games. The full model was significant (F = 11.37***, observations = 754; ** = p < .01, *** = p < .001 ).

**Supplementary Table 7**

|  | *b* | *SE* |
| --- | --- | --- |
|  |  |  |
| Intercept | -10.4*** | 2.45 |
|  |  |  |
| Fairness | 1.17*** | .133 |
|  |  |  |
| Group (LTP) | 4.35 | 3.41 |
|  |  |  |
| Fairness*Group | -.354 | .181 |

Supplementary Table 7: Beta values and standard errors from the linear mixed model using self-reported fairness evaluations of a given dictator offer and participant group (LTP versus control) to predict punishment behavior in the 3PR game. The full model was significant (F = 41.10***, observations = 350; *** = p < .001).
