# Supplementary material for: Compassion meditators show less anger, less punishment, and more compensation of victims in response to fairness violations
Source: Front Behav Neurosci. 2014 Dec 9;8:424. doi: 10.3389/fnbeh.2014.00424 (PMC4260514; doi:10.3389/fnbeh.2014.00424)
Supplement: Supplementary file 2 [file Presentation1.PDF]

Instructions for participants

You are participating in a series of studies on social decision-making. You will be interacting with other participants, each logged into a network made up of research institutions and universities across Europe dedicated to studying the nature of human social interaction. In order for you to participate and interact with the other players, we will connect you to this network.

Please read the instructions carefully. Everything that you need to know for participation in this study will be explained. In case you have any questions, please ask the experimenter.

During the course of this study you can earn points. Every point earned will be converted into Euros at the end of the experiment.

1 point = 10 cents

You will be playing multiple rounds. At the end of all rounds, one of the rounds will be randomly chosen and paid out for each participant. At the end of the study, you will be given your earnings in cash.

In the following studies, participants will be assigned different roles with different tasks and requirements. Pay close attention what these are for each study. Before each study begins, we will assign you which role you will assume.

Two Player Study

At the beginning of each round of The Two Player Study two participants, **Player A** and **Player B,** are paired with one another. Each pair interacts once only and does so anonymously.

Stage 1:

Player A receives an endowment of **100 points** and **Player B** receives an endowment of **0 points**.

Stage 2:

Player A can now decide how to **split** the endowment of **100 points** between him/herself and Player B. For this, Player A is given **six options**, whereby he can either give **50, 40, 30, 20, 10** or **0** points to Player B. These options will be displayed on the screen and Player A can make his decision by moving the mouse cursor onto the desired option and clicking with the left mouse button. Player A will see the following screen:

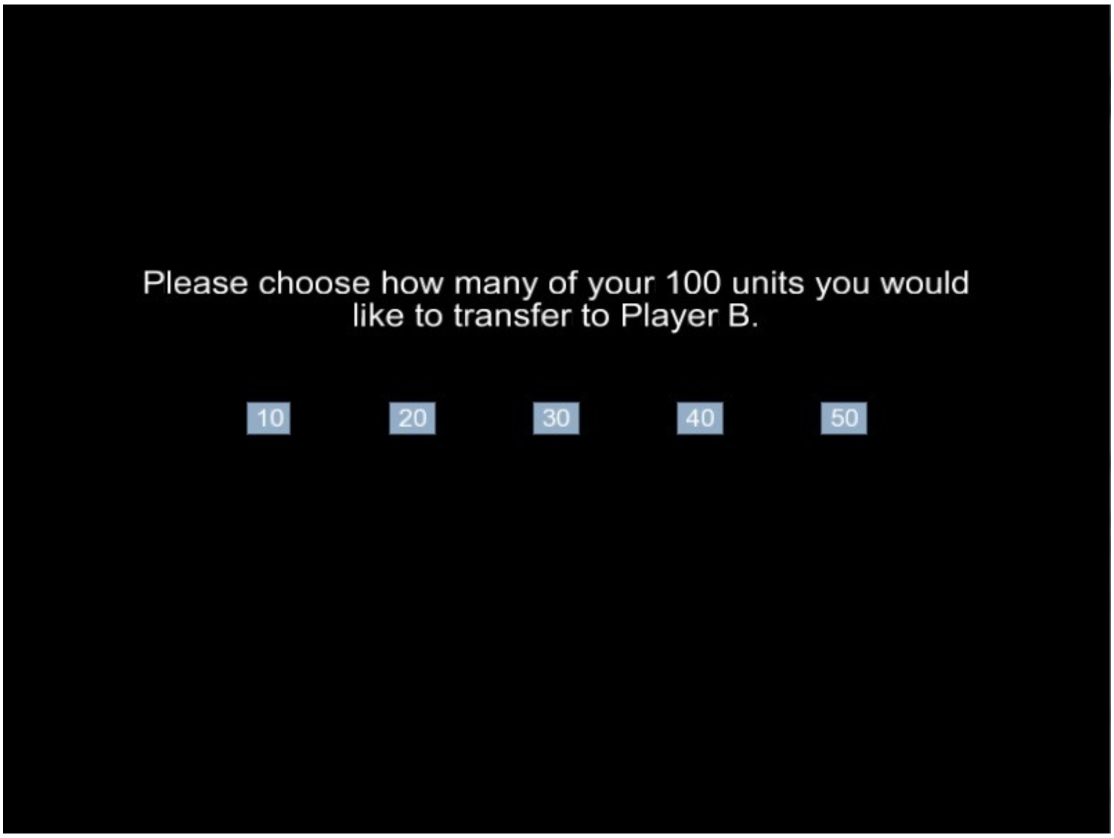

Earnings from Stage 2:

**Player A: 100 – transfer**

**Player B: transfer**

Player A transfers	Player A	Player B
**0 points**	100	0
**10 points**	90	10
**30 points**	70	30
**50 points**	50	50

Stage 3:

Now **Player B** has the opportunity to **deduct points** from the remaining endowment of **Player A**. To do so, **Player B** is given **50 points**, with which he can **pay to deduct** points from Player A. For every **1 point** Player B pays, **3 points** are deducted from **Player A**.

To do so, a slider is presented on the screen with the range of points that Player B can pay to deduct from the endowment of Player A. To make a decision, Player B must move the mouse cursor onto the slider and click with the left mouse button. Once Player B clicks on the slider, the amount he or she is paying to deduct will appear on the left below the slider. Player A's remaining endowment will appear on the right. To change the decision, Player B can simply click on the vertical bar and drag it in the slider to the desired location. It is not possible for Player A's endowment to be depleted beyond 0 points.

To finalize the decision, Player B must click on the "Submit" button at the bottom of the screen….

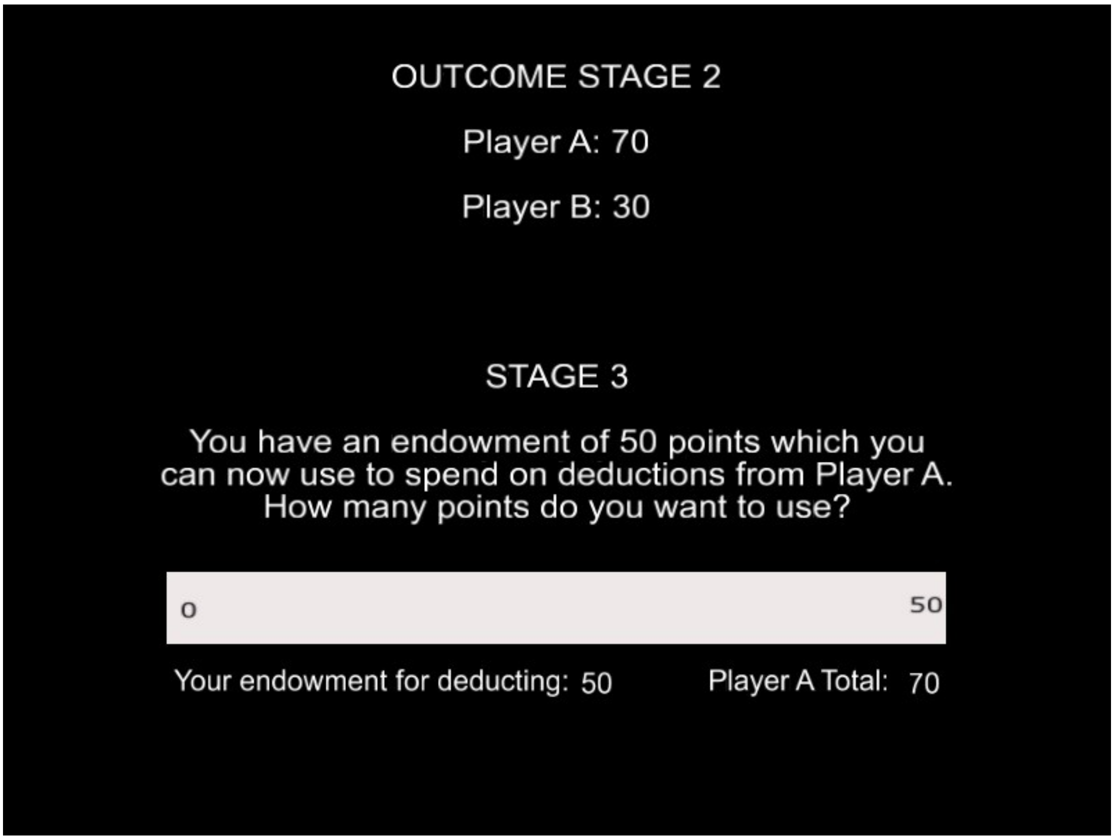
OUTCOME STAGE 2
Player A: 70
Player B: 30
STAGE 3
You have an endowment of 50 points which you can now use to spend on deductions from Player A. How many points do you want to use?
0
50
Your endowment for deducting: 50
Player A Total: 70

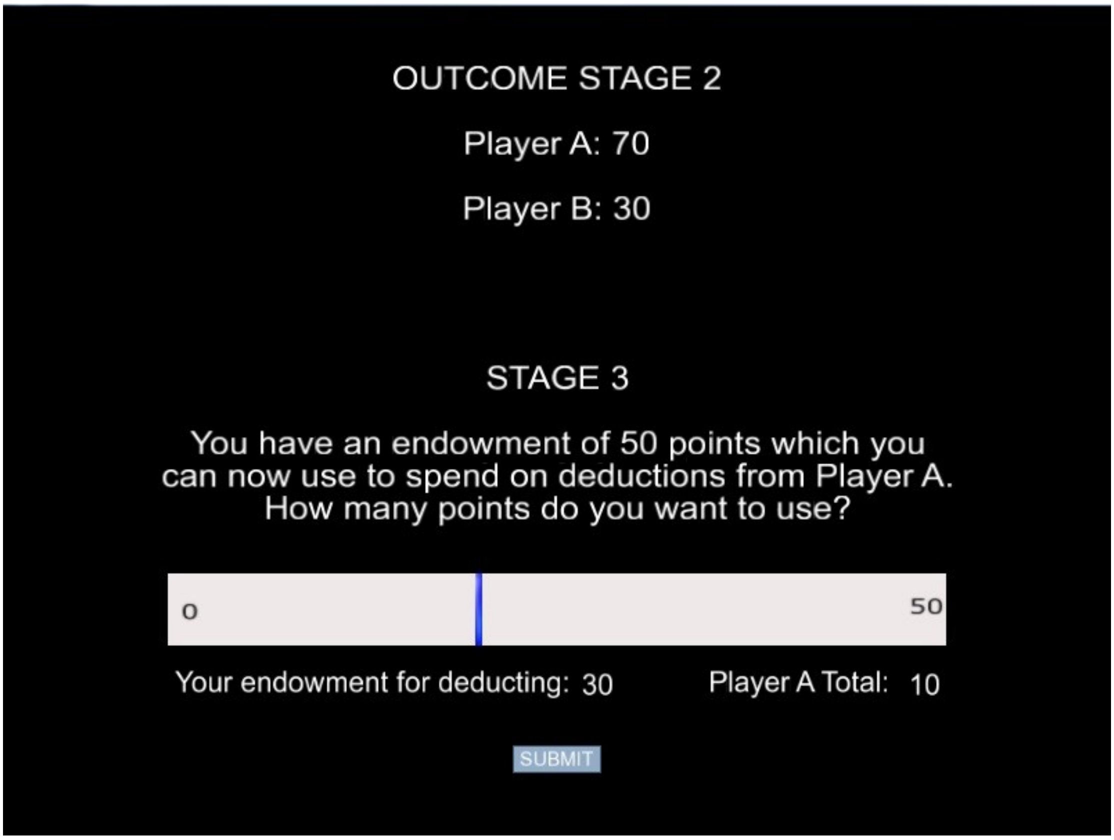
OUTCOME STAGE 2
Player A: 70
Player B: 30
STAGE 3
You have an endowment of 50 points which you can now use to spend on deductions from Player A. How many points do you want to use?
0
50
Your endowment for deducting: 30
Player A Total: 10
SUBMIT

Stage 4:

The total of Player A and Player B will be displayed on the screen. The calculations for the final points are as follows.

**Player A: 100 – transfer – (deduction points*3)**

**Player B: transfer + 50 – deduction points**

Example: For instance, Player A decides to transfer 30 points in the first stage. Player B decides to pay 20 points from his/her endowment to deduct from Player A's endowment. This leads to 60 points being deducted from Player A's remaining endowment. In this case Player A ends up with 10 points and Player B with 60 (30 from Player A's transfer and 30 from his own endowment).

Here is a table of how the final pay-off works in various circumstances.

Calculation of points depending on Player A and Player B's actions

Transfer Player A	**0**		**30**		**50**	
	**Player A**	**Player B**	**Player A**	**Player B**	**Player A**	**Player B**
Deductions Player B						
3	91	47	61	77	41	97
5	85	45	55	75	35	95
10	70	40	40	70	20	90
20	40	30	10	60	0	80

Do you have any questions?

Three Player Study I

At the beginning of each round of the Three Player Study I two participants, **Player A** and **Player B,** are paired with one another. Each pair interacts once only and does so anonymously. There is also a **Player C** who observes the interaction between the two other players.

Stage 1:

Player A receives an endowment of **100 points** and **Player B** receives an endowment of **0 points**.

Stage 2:

Player A can now decide how to **split** his endowment of **100 points** between him/herself and Player B. For this, Player A is given **six options** of how to split the endowment, whereby he can either give **50, 40, 30, 20, 10** or **0**. These options will be displayed on the screen and Player A can make his decision by moving the mouse cursor onto the desired option and clicking with the left mouse button. To finalize the decision, Player A must click on the "Submit" button at the bottom of the screen:

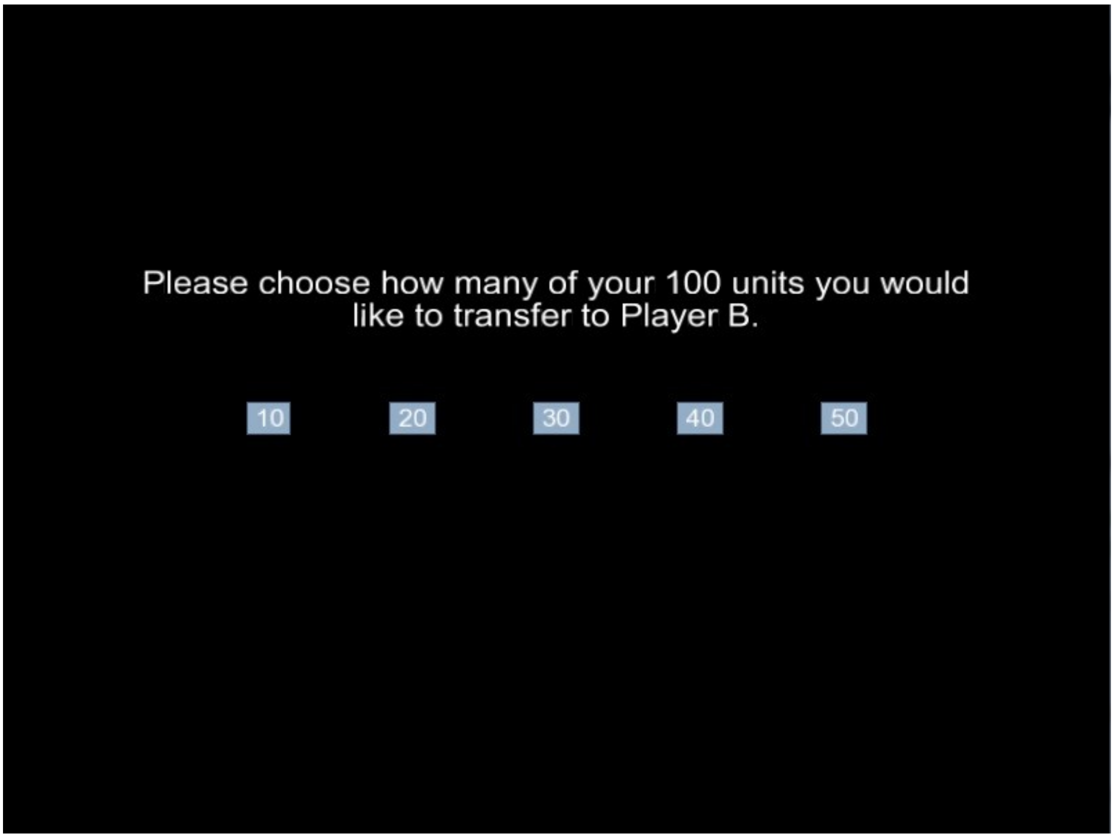

Player B will see the following screen:

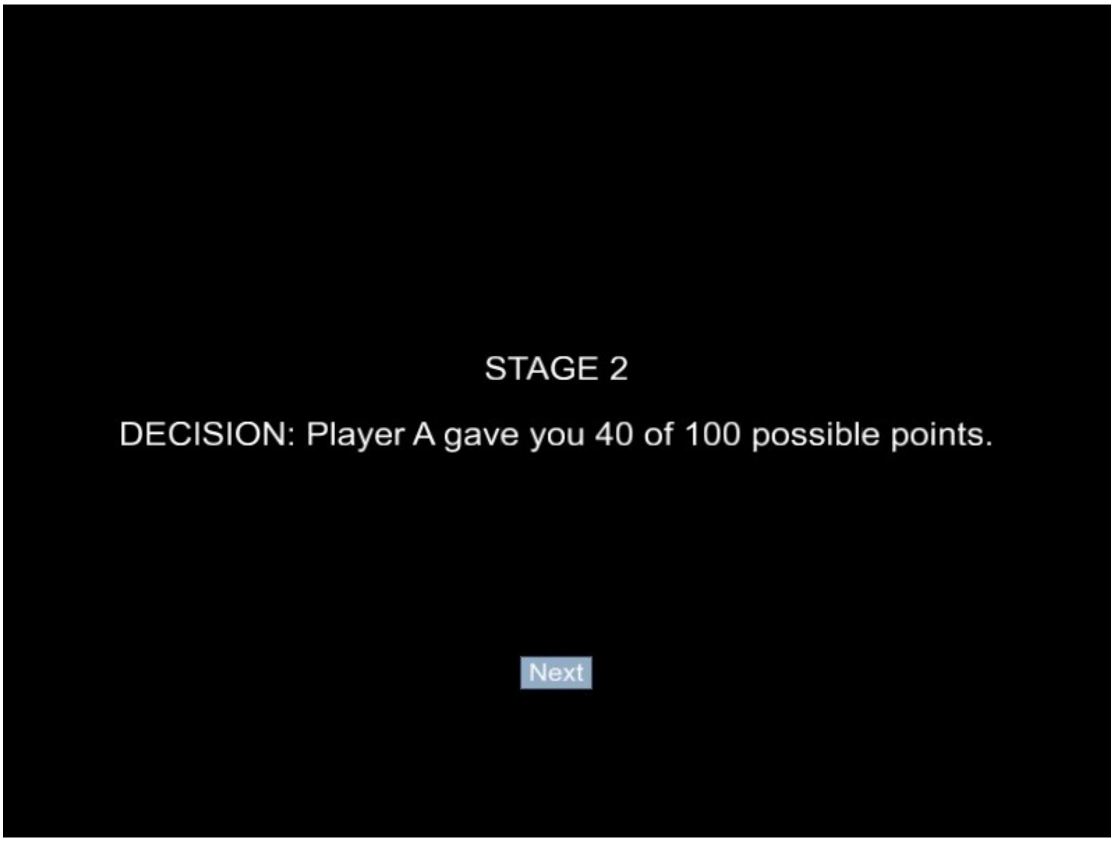

Potential earnings from Stage 2:

Player A: 100 – transfer

Player B: transfer

Player A transfers	Player A	Player B
**0 points**	100	0
**10 points**	90	10
**30 points**	70	30
**50 points**	50	50

Stage 3:

Now **Player C** has the opportunity to **deduct points** from the remaining endowment of **Player A**. To do so, **Player C** is given **50 points**, with which he can **pay to deduct** points from Player A. For every **1 point** Player C pays, **3 points** are deducted from Player A.

To do so, a slider is presented on the screen with the range of points that Player C can pay to deduct from the endowment of Player A. To make a decision, Player C must move the mouse cursor onto the slider and click with the left mouse button. Once Player C clicks on the slider, the amount he or she is paying to deduct will appear on the left below the slider. Player A's remaining endowment will appear on the right. To change the decision, Player C can simply on the vertical bar and drag it in the slider to the desired location. It is not possible for Player A's endowment to be depleted beyond 0 points.

To finalize the decision, Player C must click on the "Submit" button at the bottom of the screen….

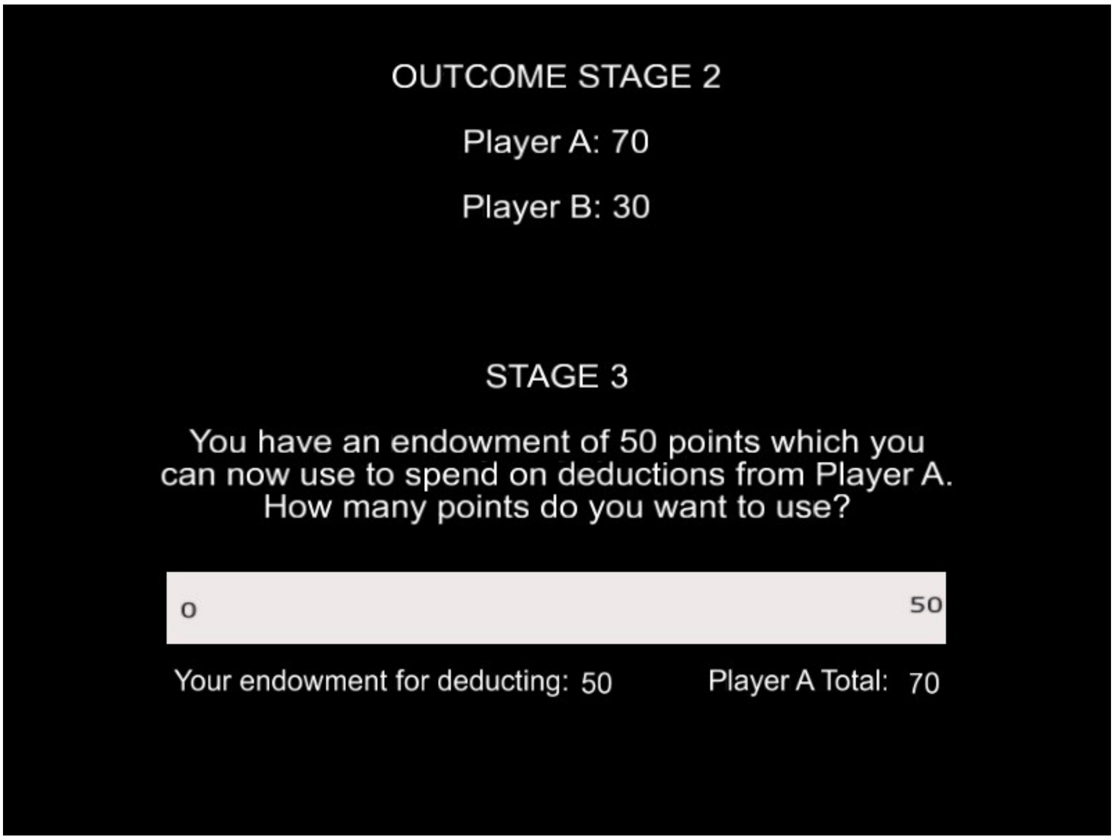

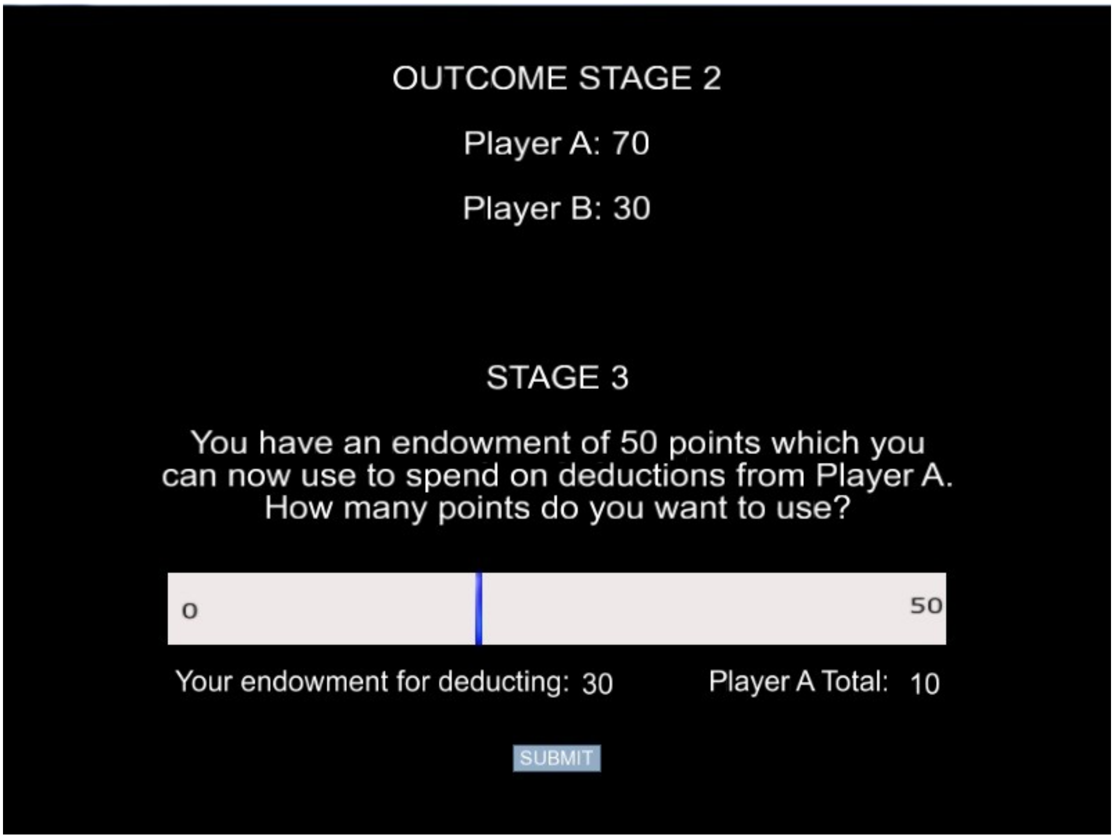

Stage 4:

The total of all players will be displayed on the screen. The calculations for the final points are as follows.

**Player A: 100 – transfer – (deduction points*3)**

**Player B: transfer**

**Player C: 50 - deduction points**

Example: For instance, Player A decides to transfer 30 points in the first stage. Player C decides to pay 20 points from his/her endowment to deduct from Player A's endowment. This leads to 60 points being deducted from Player A's endowment minus his/her transfer in the first stage. In this case Player A ends up with 10 points and Players B and C with 30.

Here is a table of how the final pay-off works in various circumstances.

Calculation of points depending on Player A and Player C's actions

Transfer Player A	**0**			**30**			**50**		
	**Player A**	**Player B**	**Player C**	**Player A**	**Player B**	**Player C**	**Player A**	**Player B**	**Player C**
Deductions Player C									
3	91	0	47	61	30	47	41	50	47
5	85	0	45	55	30	45	35	50	45
10	70	0	40	40	30	40	20	50	40
20	40	0	30	10	30	30	0	50	30

Do you have any questions?

Three Player Study II

At the beginning of each round of The Three Player Study II two participants, **Player A** and **Player B,** are paired with one another. Each pair interacts once only and does so anonymously. There is also a **Player C** who observes the interaction between the two other players.

Stage 1:

Player A receives an endowment of **100 points** and **Player B** receives an endowment of **0 points**.

Stage 2:

Player A can now decide how to **split** his endowment of **100 points** between him/herself and Player B. For this, Player A is given **six options**, whereby he can either give **50, 40, 30, 20, 10** or **0**. These options will be displayed on the screen and Player A can make his decision by moving the mouse cursor onto the desired option and clicking with the left mouse button. To finalize the decision, Player A must click on the "Submit" button at the bottom of the screen.

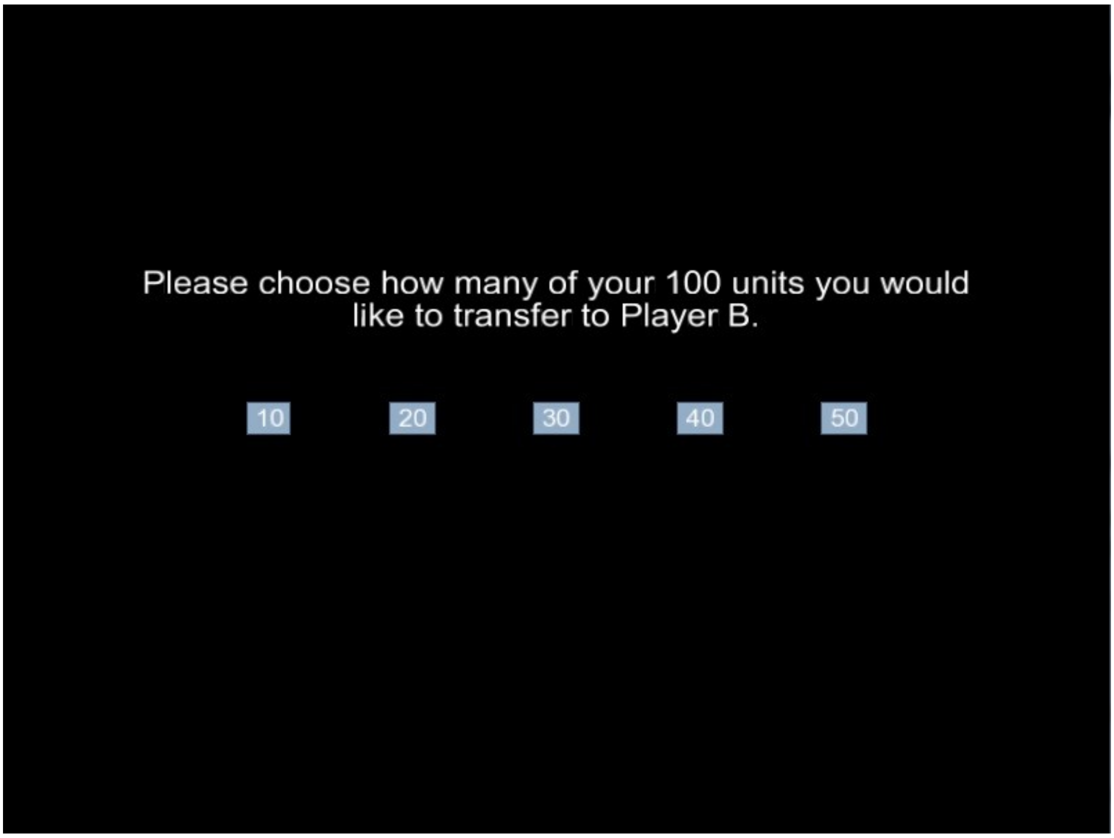

Potential earnings from Stage 2:

**Player A: 100 – transfer**

**Player B: transfer**

Player A transfers	Player A	Player B
**0 points**	100	0
**10 points**	90	10
**30 points**	70	30
**50 points**	50	50

Stage 3:

Now **Player C** has the opportunity to **simultaneously deduct or add points** from **Player A and Player B**. To do so, **Player C** is given **50 points**, with which he can **pay to deduct and add** points from Player A and Player B. For every **1 point** Player C pays, **3 points** are added or deducted.

To do so, Player C

To do so, two bars are now presented simultaneously on the screen with the range of points that Player C can pay to deduct from or add to Player A (top bar) or Player B (bottom bar). To add points to one of the two Players, Player C can click to the right of the middle of the bar, which is shaded in pale green. To deduct points from one of the two Players, Player C can click to the left of the middle of the slider, which is shaded in pale red. As soon as Player C clicks on an area on the bar, it will turn a stronger hue of the respective colour and immediately display its consequence on the pay-off of all Players. The pay-off for Players A and B is in the middle, just above their respective bar. The pay-off for Player C is written in the middle at the bottom of the screen. Note that Player C has 50 points to spend on adding and deducting. This means that with each action on one player, the range of options is increasingly limited on how to act on the other Player. The action limits are indicated by the black shading of the bar.
It is not possible for Player A's or Player B's endowment to be depleted beyond 0 points.

To finalize the decision, Player C must to click on the "Submit" button at the bottom of the screen. Player C will see the following screens:

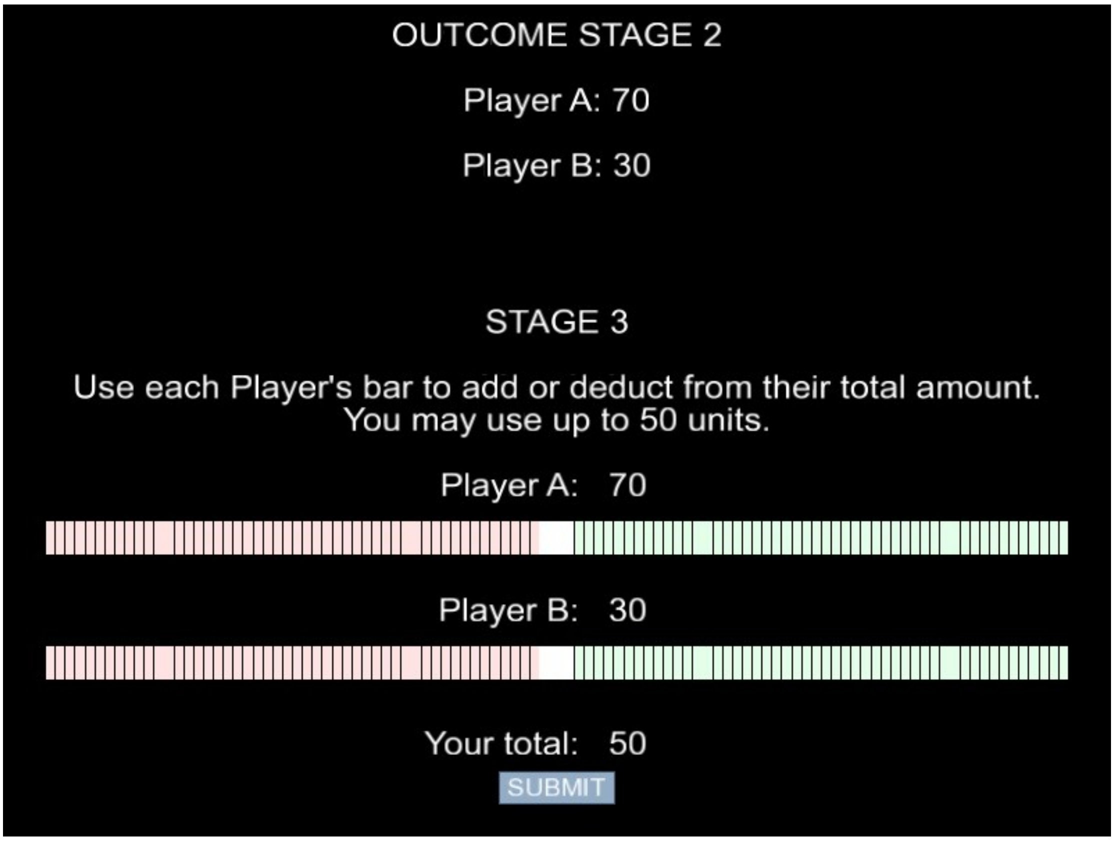
OUTCOME STAGE 2
Player A: 70
Player B: 30
STAGE 3
Use each Player's bar to add or deduct from their total amount.
You may use up to 50 units.
Player A: 70
Player B: 30
Your total: 50
SUBMIT

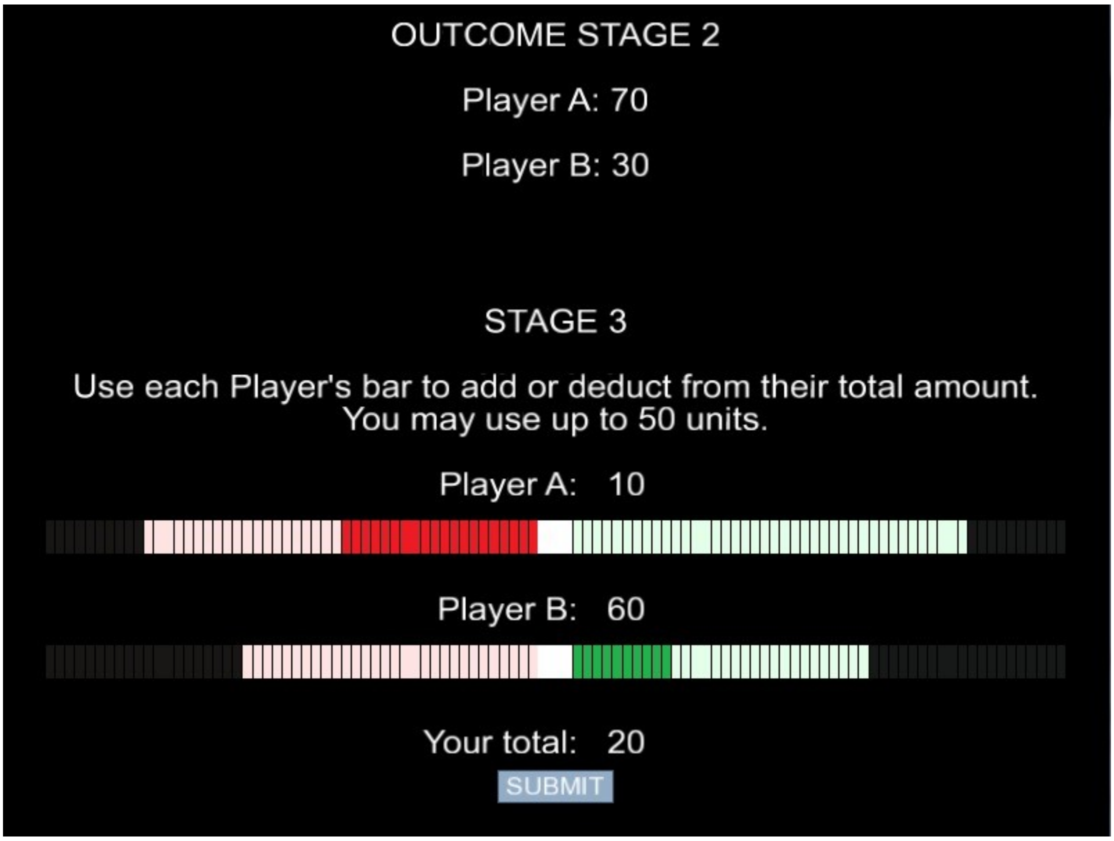
OUTCOME STAGE 2
Player A: 70
Player B: 30
STAGE 3
Use each Player's bar to add or deduct from their total amount.
You may use up to 50 units.
Player A: 10
Player B: 60
Your total: 20
SUBMIT

Stage 4:

The total of all players will be displayed on the screen. The calculations for the final points are as follows.

Player A: 100 – transfer ± (deduction / addition points*3)

Player B: transfer ± (deduction / addition points*3)

Player C: 50 – deduction / addition points

Example: For instance, Player A decides to transfer 30 points in the first stage. Player C decides to pay 20 points from his/her endowment to deduct from Player A's endowment and 10 points to add to Player B's endowment. This leads to 60 points being deducted from Player A's endowment minus his/her transfer in the first stage and 30 points being added to Player B's endowment in addition to what he received from Player A. In this case Player A ends up with 10 points, Player B with 60 and Player C with 20.

Here is a table of how the final pay-off works in various circumstances.

Calculation of points depending on Player A and Player C's actions

				Transfer Player A					
				0			50		
				Player A	Player B	Player C	Player A	Player B	Player C
Acting on Player A		Acting on Player B							
Add	Deduct	Add	Deduct						
3			3	109	0	44	59	41	44
	5	5		85	15	40	45	65	40
10		10		130	30	30	80	80	30
	20		20	40	0	10	0	0	10

Do you have any questions?
